# Ageing Is Associated with Decreases in Appetite and Energy Intake—A Meta-Analysis in Healthy Adults

**DOI:** 10.3390/nu8010028

**Published:** 2016-01-07

**Authors:** Caroline Giezenaar, Ian Chapman, Natalie Luscombe-Marsh, Christine Feinle-Bisset, Michael Horowitz, Stijn Soenen

**Affiliations:** 1 Discipline of Medicine, National Health and Medical Research Council of Australia (NHMRC) Centre of Research Excellence in Translating Nutritional Science to Good Health, the University of Adelaide, 5000 Adelaide, Australia; caroline.giezenaar@adelaide.edu.au (C.G.); ian.chapman@adelaide.edu.au (I.C.); natalie.luscombe-marsh@csiro.au (N.L.-M.); christine.feinle@adelaide.edu.au (C.F.-B.); michael.horowitz@adelaide.edu.au (M.H.); 2 Commonwealth Scientific and Industrial Research Organisation (CSIRO), Food and Nutrition, 5000 Adelaide, Australia

**Keywords:** ageing, energy intake, appetite, hunger, fullness, elderly

## Abstract

It is not well recognized that in the elderly weight loss is more common than weight gain. The aim of this analysis was to determine the effect of ageing on appetite (hunger/fullness) and energy intake, after overnight fasting and in a postprandial state, by meta-analyses of trials that included at least two age groups (>18 years). We hypothesized that appetite and energy intake would be less in healthy older compared with younger adults. Following a PubMed-database systematic search up to 30 June 2015, 59 studies were included in the random-effects-model meta-analyses. Energy intake was 16%–20% lower in older (*n* = 3574/~70 years/~71 kg/~25 kg/m^2^) than younger (*n* = 4111/~26 years/~69 kg/~23 kg/m^2^) adults (standardized mean difference: −0.77 (95% confidence interval −0.90 to −0.64)). Hunger was 25% (after overnight fasting; weighted mean difference (WMD): −17 (−22 to −13) mm) to 39% (in a postprandial state; WMD: −14 (−19 to −9) mm) lower, and fullness 37% (after overnight fasting; WMD: 6 mm (95% CI: 1 to 11 mm)) greater in older than younger adults. In conclusion, appetite and energy intake are less in healthy older than younger adults, suggesting that ageing *per se* affects food intake.

## 1. Introduction

The world population is ageing rapidly. For example, the proportion of the world’s population over 60 years will double from 11% to 22% between 2000 and 2050. As healthcare costs are incurred largely by older people, this will have dramatic societal impacts, so that, largely as a result of population ageing, it is projected that government spending in Australia on health will increase tenfold per capita by 2055 [1]. Reducing morbidity in the older population is, accordingly, a major public health goal. A very large proportion of the increases in healthcare costs are accounted for by increasing rates and duration of hospital admissions in older people. During hospitalisation, nutritional status often declines in older patients, due to a lack of adequate energy intake [2].

It is often not recognized that after age ~65 years weight loss, particularly lean tissue, is more common than weight gain—this has been well documented in cross-sectional and longitudinal studies [3,4,5,6,7,8]. In the elderly, both low body weight and weight loss are strong predictors of poor outcomes [7,9], including the development of pathological undernutrition and sarcopenia and reduced functional capacity and frailty [10]. Data form animal studies suggest that caloric restriction, and probably more importantly diet composition, play a role in longevity by reducing the risk of developing type 2 diabetes, hypertension, cardiovascular disease and cancer, which may be related to the body composition during life, *i.e.*, less fat and more lean tissue [11,12]. The loss of body weight in older people is usually associated with disproportionate loss of lean body tissue, with average decreases of up to 3 kg of lean body mass, mainly skeletal muscle, per decade after the age of ~50 years [13]. Furthermore, the adverse effects of overweight and obesity are much less in older than young adults, so that the body mass index (BMI) associated with maximum life expectancy increases with age; ~27–30 kg/m^2^ in people over 65 years compared to 20–25 kg/m^2^ in younger adults [14]. There is no sound evidence that in people over 70 years a BMI > 30 kg/m^2^ is associated with any reduction in life expectancy. Consistent with this, the lower end of the “optimum” BMI range is higher in older than young adults at about 22 kg/m^2^ [15].

Weight loss in older people occurs because there is a decrease in daily energy intake [16], which is greater than the decrease in energy expenditure [17]. The decrease in energy intake, and the reduction in appetite which underlies it, has been called the “physiological anorexia of ageing” [18,19]. The reduction in energy expenditure in the elderly is due to reduced physical exercise, loss of energy-demanding lean tissue, and decreased metabolic cost of metabolizing the smaller amount of consumed food [20,21,22]. The American National Health and Nutrition Examination Survey (NHANES) III cross sectional studies reported a decline in energy intake, between the ages of 20–29 and 70–79 years, of 38% (1138 kcal/day) in men and 27% (522 kcal/day) in women and energy intake measured with 24-h recall interviews [5]. We recently showed that energy intake was 16% lower in older than younger men, when energy intake was measured with a more accurate technique: of a single *ad libitum* buffet-style meal at the research facility [23].

An important strategy for maintaining good health in older people is the prevention and management of weight loss in the elderly. It is important, therefore, to accurately characterise this problem. Many of the studies in the area have used different methods to measure energy intake and included relatively few subjects, so there is benefit in combining these data. The aim of this analysis was to determine (i) the magnitude of decrease in energy intake and appetite by ageing; (ii) whether the age-effect on energy intake is present both after overnight fasting and in the postprandial state; and (iii) whether the age-effect on energy intake is affected by the method of energy-intake measurement, by meta-analyses of studies which included two age groups of healthy (younger and older) adults. We hypothesized that appetite and energy intake would be ~20%–25% less in healthy older when compared with younger adults.

## 2. Experimental Section

### 2.1. Search Strategy, Study Selection, Data Extraction and Quality Assessment

We performed a search of English-language publications in the PubMed database for studies that reported original data of appetite and/or energy (food) intake in “healthy” adults up to 30 June 2015. We used “ageing/aging” in combination with “appetite”, “hunger”, “fullness”, and “food/energy intake” as keywords (search terms: (“aging” (MeSH Terms) OR “aging” (All Fields) OR “ageing” (All Fields)) AND (“appetite” (MeSH Terms) OR “appetite” (All Fields)); (“aging” (MeSH Terms) OR “aging” (All Fields) OR “ageing” (All Fields)) AND (“hunger” (MeSH Terms) OR “hunger” (All Fields)); (“aging” (MeSH Terms) OR “aging” (All Fields) OR “ageing” (All Fields)) AND fullness (All Fields); (“aging” (MeSH Terms) OR “aging” (All Fields) OR “ageing” (All Fields)) AND “energy intake” (All Fields); (“aging” (MeSH Terms) OR “aging” (All Fields) OR “ageing” (All Fields)) AND “food intake” (All Fields)) with filters for animal and non-English publications. We searched for a broad and heterogeneous range of studies, and not only intervention studies, reporting data on appetite and energy intake in both younger and older adults. These data are often reported as “subject characteristics” at baseline, particularly in the case of energy intake, and not as primary study outcomes. Two researchers (CG and SS) performed screening of studies by titles and abstracts and, subsequently, full texts. References from the retrieved publications and bibliographies of relevant reviews were checked to identify potential additional articles. Studies were included if they reported mean ± SD/SEM energy intake (kcal) and/or appetite (*i.e.*, hunger and/or fullness) of at least two age groups—“younger” and “older” adults. Study subjects were required to be “healthy” and at least 18 years old, without using age restrictions in defining the “younger” and “older” age groups. Usually the older groups were made up of people over 60–65 years. Animal studies and non-English publications were excluded. Characteristics were extracted from the original reports using a standardized data extraction form. When SD’s or SEM’s of appetite or energy intake were missing in the publication or it was stated that these data were measured but not given, the investigators were contacted by e-mail with a request to provide these data—requested and received twice regarding data of appetite and once requested but not received regarding data of energy intake. We recorded the study’s author(s), year of publication, study design, number and gender distribution of the participants, and mean ± SD: age (years), body weight (kg), body mass index (BMI, kg/m^2^) for both age groups (Table 1). The usual quality filters for randomized trials or observational epidemiologic studies did not apply since the primary aim of this meta-analysis was to determine the magnitude of decrease in energy intake and appetite by ageing rather than to determine the effect of an intervention. We determined whether studies reported inclusion and exclusion criteria and data on attrition, and whether potential confounders were considered, for example whether the younger and older groups were matched for body weight and/or BMI. When data of interventions were used, we reported whether randomization of study conditions was used and whether the study subjects and research personnel were blinded. This meta-analysis is reported in accordance with the recommendations and criteria outlined in the Preferred Reporting Items for Systematic Reviews and Meta-Analysis (PRISMA) statement [24].

### 2.2. Data Analysis

Meta-analyses were performed with REVMAN software (Version 5.2; the Cochrane Collaboration Oxford, UK) using the DerSimonian and Laird random-effects model with a 95% confidence interval, to account for measurement variability among the included studies. For this analysis, the number of participants’ means and SD’s of energy intake and hunger and fullness were extracted for both age groups, *i.e.*, younger and older adults. For all data, the SD’s were calculated, when necessary, from SE’s, and when data were not provided in numerical form they were estimated from the figures. Cochran’s test for heterogeneity was used to determine whether the studies included in the meta-analysis were evaluating the same underlying sizes of effect. A threshold of *p* < 0.1 was used to decide whether heterogeneity (genuine variation in effect sizes) was present. *I*^2^, an estimate of the proportion of total observed variability that is due to genuine variation rather than random error within studies, was used to quantify the degree of inconsistency among studies; it was considered substantial when it was >50% [25]. Sensitivity analyses were performed on studies that may cause bias in the results. Differences in energy intake between younger and older adults were analysed using standardized mean differences (SMD’s). The SMD is used when it is necessary to standardize the results of several studies to a uniform scale—when studies assess the same outcome (e.g., energy intake) but measure it in a variety of ways (e.g., kcal/meal or kcal/day for energy intake). The SMD expresses the size of the effect in each study relative to the variability observed in that study. The SMD is calculated by dividing the difference in mean outcome between groups (younger and older adults) by the SD of outcome among participants [25]. Data relating to hunger and fullness were defined as mean difference between the younger and older adults. Percentage differences between the younger and older adults for the outcomes were calculated for each study and averaged.

**Table 1:** Studies included in the meta-analysis.

Study (No in References)	N Young/Older	Age (Years) Young/Older	Mean Body Mass (kg) Young/Older	Mean BMI (kg/m^2^) Young/Older	Outcomes Used for Meta-Analysis
Alam *et al.* 2012 [26]	131/526	34 ± 9/69 ± 6	62.4 ± 13.5/63.5 ± 10.2 †	23.2 ± 2.2/22.3 ± 1.7 †	Energy intake of 24-h food intake recalls
Apolzan *et al.* 2009 [27]	24/32	25 ± 5/71 ± 6	75.5 ± 21.1/74.1 ± 18.7 †	25.2 ± 3.9/26.0 ± 5.1 †	Energy intake of 24-h food intake recalls
Arciero *et al.* 2009 [28]	0 M; 10 F/0 M; 10 F	19 ± 2/55 ± 5	62.5 ± 7.3/72.1 ± 9.4 *		Energy intake of 3-day weighed food records
Bell *et al.* 2003 [29]	7 M; 5 F/12 M; 9 F	23 ± 3/68 ± 5	70.4 ± 11.8/77.2 ± 13.7 †	23.7 ± 2.4/26.6 ± 3.7 *	Energy intake of 4-day weighed food records
Cheng *et al.* 1978 [30]	8 M; 0 F/7 M; 0 F	26 ± 3/67 ± 5	66.5 ± 7.2/61.6 ± 11.3 †		Energy intake of weighed food records
Church *et al.* 1984 [31]	7 M; 8 F/6 M; 8 F	20-35/36-53	45.0-95.3/52.6-85.4		Energy intake of weighed food records
Clarkston *et al.* 1997 [32]	10 M; 9 F/5 M; 9 F	30 ± 35/76 ± 19		25.3 ± 3.4/25.2 ± 1.7 †	Hunger/fullness during fasting and postprandial (456 kcal oral mixed nutrient preload) conditions
Cook *et al.* 1997 [33], MacIntosh *et al.* 1999 [34] ^#^	7 M; 0 F/8 M; 0 F	27 (20–34)/70 (65–75)		26.8 (24.4–31.8)/25.8 (18.2–30) †	- Energy intake of 5-day weighed food records - Energy intake during postprandial conditions ‡ (348 kcal intraduodenal lipid infusion) - Hunger/fullness during fasting conditions
Davy *et al.* 2001 [35]	6 M; 0 F/5 M; 0 F	25 ± 2/63 ± 7	79.0 ± 7.3/82.0 ± 8.9 †		Energy intake of 4-day weighed food records
Di Francesco *et al.* 2010 [36]	6 M; 6 F/5 M; 7 F	28 ± 2/75 ± 6		18.9–26.5/21.1–28.3 †	Hunger during fasting and postprandial (800 kcal oral mixed nutrient preload) conditions ^
Di Francesco *et al.* 2006 [37]	4 M; 4 F/4 M; 4 F	30 ± 3/78 ± 3		22.7–25.7/22.1–29.4 †	Hunger/fullness during fasting and postprandial (800 kcal oral mixed nutrient preload) conditions
Di Francesco *et al.* 2005 [38]	5 M; 4 F/5 M; 5 F	32 ± 8/77 ± 3		22.7–28.1/23.5–29.3	Hunger/fullness during fasting and postprandial ‡ (800 kcal oral mixed nutrient preload) conditions
Drewnowski *et al.* 1996 [39]	12 M; 12 F/12 M; 12 F	23 ± 1/67 ± 2		22.7 ± 1.0/24.5 ± 1.2	Energy intake of 14-day weighed food records
Flint *et al.* 2008 [40]	16 M; 14 F/16 M; 14 F	25 ± 4/68 ± 5	71.0 ± 10.4/73.8 ± 17.0 †	24.6 ± 2.2/24.7 ± 2.2 †	Energy intake of 4-day weighed food records
Fukagawa *et al.* 1990 [20]	6 M; 0 F/6 M; 0 F	21 ± 2/72 ± 7			Energy intake of 14-day dietary recalls
Giada *et al.* 1995 [41]	24 M; 0 F/24 M; 0 F	24 ± 4/57 ± 6		23.7 ± 2.4/26.8 ± 2.5 †	Energy intake of 7-day weighed food records
Howarth *et al.* 2007 [42]	1021 M; 771 F/491 M; 402 F	39 ± 17/71 ± 12		25.2 ± 4.2/25.4 ± 6.0 †	Energy intake of 24-h food intake recalls
Ishikawa *et al.* 1999 [43]	53 M; 16 F/50 M; 32 F	30-49/50-69	69.2 ± 10.4/62.9 ± 8.6	25.2 ± 3.0/25.0 ± 2.7	Energy intake of 2-day weighed food records
Keene *et al.* 1998 [44]	7 M; 5 F/4 M; 6 F	25/75			Energy intake during postprandial conditions ‡ (447 kcal oral mixed nutrient preload)
Kos *et al.* 1996 [45]	0 M; 38 F/0 M; 17 F	29 ± 3/59 ± 4	61.6 ± 9.7/57.4 ± 8.3 †	21.7 ± 3.1/21.8 ± 2.8 †	Energy intake of 4-day weighed food records
Lieberman *et al.* 1989 [46]	21 M; 20 F/21 M; 24 F	26 (20–35)/73 (65–95)			Energy intake of 4-day weighed food records
Macintosh *et al.* 2001 [47]	5 M; 7 F/5 M; 7 F	23 (20-26)/72 (65-84)		24.7 ± 2.4/25.0 ± 1.7 †	- Energy intake during fasting conditions ‡ - Energy intake of 3-day weighed food records - Hunger/fullness during fasting conditions
Macintosh *et al.* 2001 [48]	6 M; 6 F/6 M; 6 F	23 ± 4/71 ± 5		23.5 ± 2.8/24.1 ± 2.4 †	- Energy intake during fasting conditions ‡ - Energy intake of 3-day weighed food records - Hunger during fasting conditions
Macintosh *et al.* 2001 [49]	13 M; 0 F/13 M; 0 F	24 ± 5/72 ± 6		23.9 ± 2.2/23.5 ± 3.6 †	- Energy intake during postprandial conditions ‡ (347 kcal intraduodenal lipid infusion) - Energy intake of 3-day weighed food records - Hunger/fullness during postprandial conditions ‡ (347 kcal intraduodenal lipid preload)
McGandy *et al.* 1966 [50]	13 M; 0 F/37 M; 0 F	20-34/75-99	74.5 ± 1.2/70.9 ± 1.0		Energy intake of 7-day weighed food records
Morais *et al.* 2000 [51]	4 M; 3 F/3 M; 5 F	28 ± 5/72 ± 3	63.5 ± 10.6/64.2 ± 10.2 †	21.4 ± 2.1/24.8 ± 3.1 *	Energy intake of 6-day weighed food records
Morais *et al.* 1997 [52]	8 M; 7 F/8 M; 8 F	28 ± 5/73 ± 5	62.6 ± 7.4/64.1 ± 8.7 †	21.2 ± 1.8/23.8 ± 3.2 *	Energy intake of 6-day weighed food records
Moriguti *et al.* 2000 [53]	5 M; 6 F/9 M; 9 F	26 ± 3/68 ± 3	65.6 ± 9.6/80.0 ± 14.9 *	23.2 ± 1.6/27.5 ± 3.4 *	Energy intake of provided food items (7 days)
Nagengast *et al.* 1988 [54]	5 M; 6 F/6 M; 5 F	22 ± 6/67 ± 5	67.6 ± 5.0/69.1 ± 12.3 †		Food intake recalls
Poehlman *et al.* 1990 [55]	42 M; 0 F/26 M; 0 F	25 ± 5/67 ± 5	75.5 ± 10.7/78.4 ± 7.6 †		Energy intake of 3-day weighed food records
Polito *et al.* 2005 [56]	48 M; 47 F/103 M; 96 F	61 ± 4/74 ± 4	71.5 ± 8.1/67.9 ± 9.0	26.1 ± 2.4/25.3 ± 2.7	Energy intake of 4-day weighed food records
Rayner *et al.* 2000 [57]	5 M; 0 F/5 M; 0 F	23 (22–27)/71 (68–73)		24.4 (20.7–31.2)/25.6 (22.4–30.7)	- Energy intake during fasting conditions ‡ - Hunger/fullness during fasting conditions ^
Roberts *et al.* 1996 [58]	7 M; 0 F/9 M; 0 F	24 ± 1/70 ± 7	76.2 ± 12.4/72.9 ± 9.3 †	23.9 ± 3.4/23.4 ± 3.3 †	Energy intake of provided food items (10 days)
Roberts *et al.* 1994; 1995 [22,59] ^#^	17 M; 0 F/18 M; 0 F	23 ± 2/68 ± 6	71.6 ± 11.1/78.8 ± 12.6	23.4 ± 2.6/25.2 ± 3.6	Energy intake of provided food items (7 days)
Rolls *et al.* 1995 [60]	16 M; 0 F/16 M; 0 F	24 ± 5/69 ± 6	74.0 ± 7.2/84.3 ± 12.8 *	22.7 ± 2.0/26.2 ± 3.6 *	- Energy intake during fasting and postprandial (510 kcal oral mixed nutrient preload) conditions - Hunger/fullness during fasting and postprandial ‡ (510 kcal oral mixed nutrient preload) conditions
Rolls *et al.* 1991 [61]	12 M; 12 F/12 M; 12 F	26 ± 4/75 ± 5	68.9 ± 3.0/66.0 ± 3.2	23.5 ± 3.0/24.1 ± 2.8	Hunger/fullness during fasting conditions
Sawaya *et al.* 2001 [62]	9 M; 0 F/10 M; 0 F	23 ± 1/69 ± 1	72.9 ± 2.7/74.7 ± 3.4 †	22.7 ± 0.5/24.4 ± 0.9 †	Hunger during fasting conditions
Sawaya *et al.* 1996 [63]	0 M; 10 F/0 M; 10 F	25 ± 4/74 ± 4	54.8 ± 4.1/58.7 ± 9.8 †	20.9 ± 1.9/24.1 ± 2.8 *	Energy intake of 7-day weighed food records
Schneider *et al.* 2008 [64]	5 M; 5 F/3 M; 6 F	34 ± 8/76 ± 9		22.5 ± 2.9/23.6 ± 1.8 †	Hunger/fullness during fasting conditions ‡
Serra-Prat *et al.* 2013 [65]	7 M; 12 F/13 M;7 F	38 ± 11/81 ± 8	67.3 ± 9.0/72.6 ± 16.2	23.7 ± 2.8/27.9 ± 4.9 †	- Hunger during fasting and postprandial ‡ (400 kcal oral mixed nutrient preload) conditions
Serra-Prat *et al.* 2009 [66]	7 M; 10 F/6 M; 4 F	40 ± 10/80 ± 8		25.2 ± 3.3/26.7 ± 3.0 †	- Hunger during fasting and postprandial ‡ (380 kcal oral mixed nutrient preload) conditions
Soenen *et al.* 2014 [23]	10 M; 0 F/10 M; 0 F	23 ± 4/74 ± 4	73 ± 7/79 ± 7 †	22 ± 2/26 ± 2 *	- Energy intake during fasting and postprandial (180 kcal intraduodenal protein infusion) - Hunger/fullness during fasting and postprandial (180 kcal intraduodenal protein infusion) conditions
Stafleu *et al.* 1994 [67]	0 M; 97 F/0 M; 97 F	25 ± 3/76 ± 6	64.2 ± 10.6/70.5 ± 10.7	22.5 ± 3.5/26.8 ± 4.1	Energy intake of food frequency questionnaires
Sturm *et al.* 2004 [68]	6 M; 6 F/6 M; 6 F	24 ± 1/74 ± 1		23.2 ± 2.1/24.1 ± 3.5 †	- Energy intake during fasting and postprandial ‡ (750 kcal oral mixed nutrient preload) conditions - Energy intake of 3-day weighed food records - Hunger/fullness during fasting and postprandial ‡ (750 kcal oral mixed nutrient preload) conditions
Sturm *et al.* 2003 [69]	0 M; 8 F/0 M; 8 F	22 ± 4/77 ± 3	57.5 ± 5.4/58.0 ± 5.9 †	20.5 ± 1.1/23.7 ± 2.3 *	- Energy intake during fasting and postprandial (280 kcal oral mixed nutrient preload) conditions - Energy intake of 3-day weighed food records - Hunger/fullness during fasting and postprandial (280 kcal oral mixed nutrient preload) conditions ‡
Surrao *et al.* 1998 [70]	0 M; 10 F/0 M; 10 F	25 ± 4/74 ± 4	54.8 ± 4.1/58.7 ± 9.8 †	20.9 ± 1.9/24.1 ± 2.5 *	Energy intake of a 7-day weighed food record
Temme *et al.* 2010 [71]	413 M; 460 F/389 M; 355 F				Energy intakes of 24-h food intake recalls and food frequency questionnaires
Toth *et al.* 1996 [72]	18 M; 0 F/30 M; 0 F	23 ± 4/69 ± 5	79 ± 8/75 ± 5 †		Energy intake of 3-day weighed food records
Van Pelt *et al.* 2001 [73]	71 M; 0 F/66 M; 0 F	27 ± 8/62 ± 8	75.1 ± 16.0/77.4 ± 16.2	23.4 ± 4.7/25.1 ± 4.4	Energy intake of 4-day weighed food records
Van Walleghen *et al.* 2007 [74]	14 M; 15 F/11 M; 10 F	25 ± 5/69 ± 9	67.9 ± 1.7/70.8 ± 2.9	23.3 ± 3.7/24.7 ± 3.2	- Energy intake during fasting conditions ‡ - Energy intake of 4-day weighed food records - Hunger/fullness during fasting conditions
Van Walleghen *et al.* 2007 [75]	14 M; 15 F/13 M; 12 F	24 ± 5/68 ± 10	67.6 ± 15.5/71.1 ± 16.5	23.3 ± 4.3/24.6 ± 3.8	- Energy intake during fasting and postprandial (476 kcal for males and 360 kcal for females oral mixed nutrient preloads) conditions - Energy intake of 4-day weighed food records - Hunger/fullness during fasting and postprandial (476 kcal for males and 360 kcal for females oral mixed nutrient preload) conditions ‡ - Hunger/fullness during fasting conditions
Vaughan *et al.* 1991 [21]	33 M; 31 F/17 M; 21 F	24 ± 4/71 ± 6	84.5 ± 23.1/71.2 ± 13.5 *		Energy intake of provided food items (1 day)
Winkels *et al.* 2010 [76]	15 M; 0 F/17 M; 0 F	24 (20–34)/68(64–85)	75.8 ± 11.3/75.8 ± 7.6	23.0 ± 2.3/24.5 ± 1.9	Energy intake of provided food items (14 days)
Wolk *et al.* 2004 [77]	72 M; 0 F/94 M; 0 F	42–54/65–76		25.6 ± 2.7/26.5 ± 3.7	Energy intake of 24-h food intake recalls
Wright *et al.* 1995 [78]	41 M; 42 F/28 M; 43 F	20–64/74–90	70.1 ± 10.4/64.8 ± 10.1		Energy intake of 7-day weighed food records
Wurtman *et al.* 1988 [79]	21 M; 20 F/21 M; 24 F	26 (19–35)/72 (65–94)			Energy intake of provided food items (5 days)
Yukawa *et al.* 2006 [80]	8 M; 13 F/7 M; 11 F	25 ± 5/75 ± 4	72.9 ± 12.4/73.6 ± 12.7 †	24.7 ± 3.0/26.9 ± 3.0 *	Energy intake of provided food items (14 days)
Zandstra *et al.* 2000 [81]	5 M; 28 F/6 M; 18 F	22 ± 2/76 ± 5	71.0 ± 9.6/72.4 ± 8.9	23.3 ± 2.3/26.6 ± 3.5	Energy intake during fasting and postprandial (502 kcal for young subjects) or 430 kcal for older subjects oral mixed nutrient preload) conditions ‡
Zhou *et al.* 2013 [82]	49 M; 10 F/15 M; 21 F	20–29/50–59	59.0 ± 10.8/69.0 ± 12.3	21.7 ± 3.0/24.6 ± 3.1	Fullness during fasting and postprandial (896 kcal oral mixed nutrient preload) conditions

M, male; F, female; BMI, body mass index; All values are mean ± SD (range; when SD was not reported); ^#^ Studies reported same data; * *p* < 0.05, older compared with young; † *p* > 0.05, older compared with young, When blank the significance of the comparison of body weight and/or BMI between older and young adults had not been reported; ‡ Data were extracted from graphs, by creating a scale according to the *y*-axis, and measuring mean and SD/SEM with a ruler; ^ Data were provided by the investigators upon request.

## 3. Results

**Figure 1:**
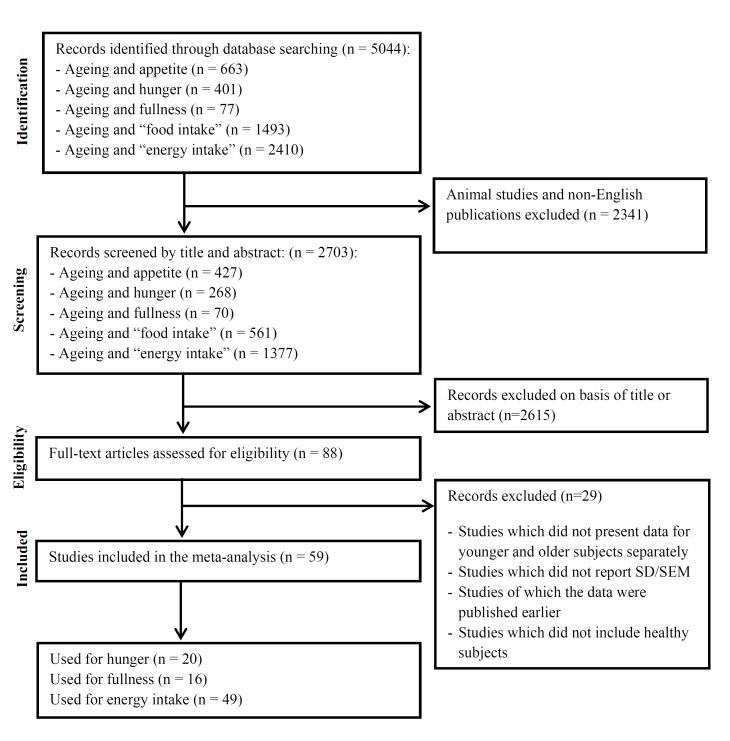
Flow diagram for the selection of studies.

The Pubmed search identified 5044 potential articles. The review flow diagram is, following the recommendations of the PRISMA statement [24], depicted in Figure 1. We screened 2703 titles or abstracts following exclusion of 2341 animal studies or non-English articles. We screened 88 publications in full text of which 59 studies fulfilled the inclusion criteria. There were seven studies that included more than two age groups [54,61,67,71,77,78,81]—we extracted the data of energy intake and/or appetite of the youngest (≥18 years) and oldest age group for each of these studies. There were 14 studies that presented data of multiple groups within the younger and older study groups [31,39,41,43,46,52,55,56,73,74,75,78,79,83], *i.e.*, gender, country, and/or level of physical activity—we combined the male and female or country or level of physical activity groups by calculating their mean energy intake/appetite score and pooling their SD’s to create a single pair-wise comparison.

No studies were excluded based on quality of study, although many studies did not report sufficient information for a clear bias assessment. All studies, except four [26,45,46,50], reported inclusion and/or exclusion criteria, and stated that the participants met these criteria. Of the studies measuring energy intake (49 studies), 18 studies matched the younger and older participants for body weight [23,26,27,29,30,35,40,45,51,52,54,58,63,69,70,72,76,80] and 13 studies for BMI [26,27,33,40,41,42,45,47,48,49,58,68,76] and 11 studies considered gender as a confounder [31,39,43,46,47,52,56,71,75,78,79]. No studies considered confounders for hunger or fullness.

In crossover studies (17 studies [23,33,36,44,47,48,49,57,60,61,62,68,69,74,75,81,82]), selection bias and performance bias were possible sources of bias. Thirteen studies [23,33,47,48,49,57,60,68,69,74,75,81,82] were randomized, of which one [23] detailed a method through random numbers, in the other six, randomization was not discussed. No studies reported the use of allocation concealment. Performance bias scored worse, three studies were double-blind [23,47,60] and two single-blind [44,49], in the other 12 blinding was not discussed. In the studies measuring energy intake over a prolonged period of time, all studies, except two [58,59], had a method to check compliance.

### 3.1. Effect of Age on Energy Intake

Three different methods of measuring energy intake were distinguished: (i) energy intake of a single *ad libitum* buffet-style meal at the research facility after overnight fasting (during a “control” condition; e.g., no preload, water preload or saline intraduodenal infusion [23,47,48]) and in a postprandial state after a nutrient preload (>0 kcal; range 180 [23]—729 [81] kcal), administered orally or infused directly into the small intestine (*i.e.*, intraduodenally [23,49])—all intervention crossover studies; (ii) energy intake of provided food items during a prolonged period (~4 days–2 weeks); and (iii) energy intake of weighed food records (~3–14 days), 24-h food intake recalls, or food frequency questionnaires – we used the observational data for energy intake for the latter two categories. There were three studies measuring energy intake of a buffet-style meal in a postprandial state which consisted of multiple nutrient-preload conditions [23,49,60]—we extracted the energy intake data of the condition which had the largest effect to suppress energy intake by the nutrient ingestion in the younger-subject group (*i.e.*, the nutrient preload with the highest energy content).

Forty-nine unique studies presented data on energy intake: (i) 10 studies (311 subjects) reported energy intake of “a buffet-style meal” after overnight fasting [23,47,48,57,60,68,69,74,75,81] and nine studies (266 subjects) in a postprandial state [23,33,44,49,60,68,69,75,81]—of which six studies (203 subjects) measured energy intake both after overnight fasting and in a postprandial state [23,60,68,69,75,81]; (ii) seven studies (339 subjects) reported energy intake “during a prolonged period” [21,53,58,59,76,79,80]; and (iii) 37 studies (7035 subjects) reported energy intake of weighed-food records (30 studies [20,28,29,30,31,34,35,39,40,41,43,45,46,47,48,49,50,51,52,55,56,63,68,69,70,72,73,74,75,78], 24-h food intake recalls (six studies [26,27,42,54,71,77]), or food frequency questionnaires (one study [67])—of which eight studies also reported energy intake of a buffet-style meal [33,47,48,49,68,69,74,75].

Twenty-six studies were conducted in the United States [20,21,27,28,29,31,35,39,40,42,46,50,53,55,58,59,60,62,63,70,72,73,74,75,78,79,80], 10 in Europe [41,44,45,54,56,67,71,76,77,81], eight in Australia [23,34,47,48,49,68,69], two in Asia [26,43], two in Canada [51,52], and one in Chile [30]. The oldest study was published in 1966 [50] and the most recent in 2014 [17]. The largest study included 2685 subjects [42] and the smallest 10 subjects [57]. The mean age of the youngest group within a study was 19 years (the older group in that study had a mean age of 55 years) [28] and of the oldest group 77 years (the younger group in that study had a mean age of 22 years) [69].

#### 3.1.1. Energy Intake in the Total Group

**Figure 2:**
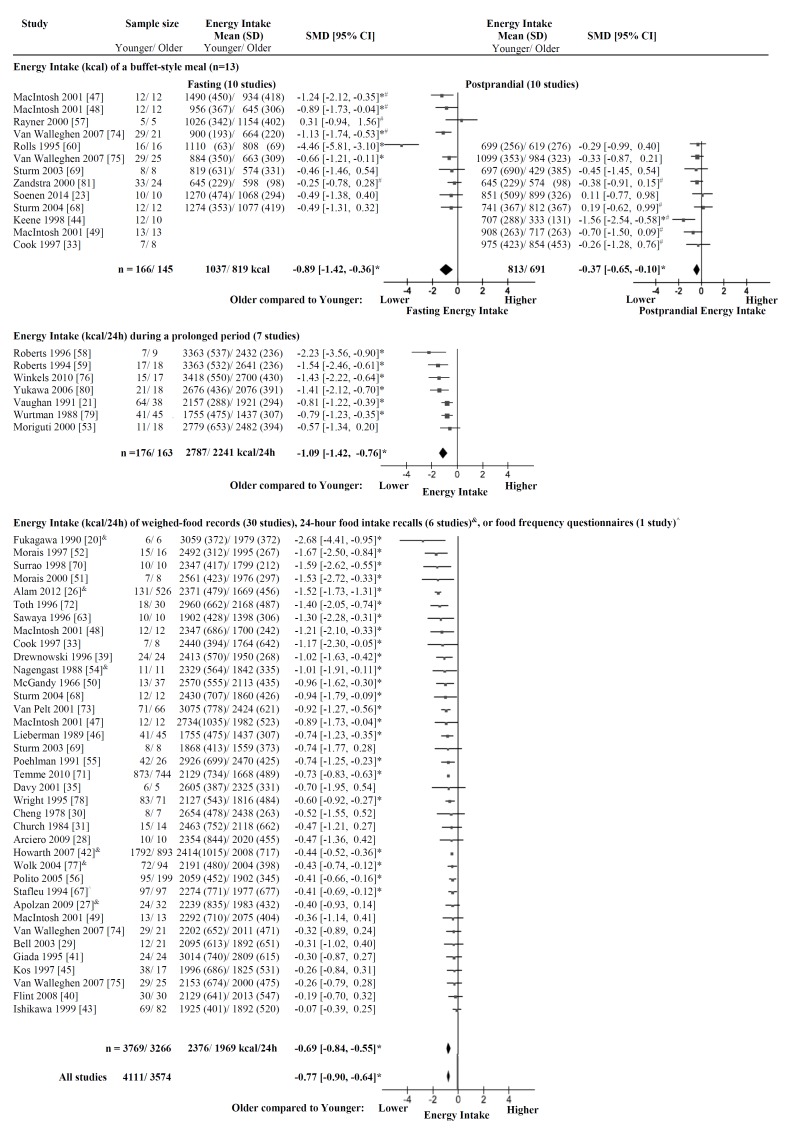
Energy intake. Mean ± SD of energy intake (kcal) and a plot of the standardized mean difference (SMD; mm) of energy intake in older compared with younger subjects with the DerSimonian and Laird random-effect model. The horizontal lines denote the 95% confidence interval; ▀ point estimates (the size of the square corresponds to its weight); ♦ the pooled estimate of age effect. Three different methods of measuring energy intake were distinguished: (i) energy intake of a single *ad libitum* buffet-style meal at the research facility after overnight fasting and in a postprandial state after a nutrient preload, administered orally or infused directly into the small intestine; (ii) energy intake of provided food items during a prolonged period; and (iii) energy intake of weighed food records, 24-h food intake recalls, or food frequency questionnaires. In the total group of 7685 subjects, energy intake was less (SMD: −0.77 (95% CI −0.90 to −0.64), *I*^2^ = 76%, *p* < 0.001) in the older than the younger adults. * *p* < 0.05 energy intake significantly less in older than younger adults within the study; ^#^ data were derived from a figure of the original publication; food records, ^&^ 24-h food intake recalls; or ^ food frequency questionnaires.

In the total group of 7685 subjects, energy intake after overnight fasting was less in the older (*n* = 3574, ~70 years, body weight ~71 kg, BMI ~25 kg/m^2^) than the younger adults (*n* = 4111, ~26 years, ~69 kg, ~23 kg/m^2^), with a SMD of −0.77 (95% CI: −0.90 to −0.64) (Figure 2) and significant heterogeneity (*I*^2^ = 76%, *p* < 0.001). As a group, the older adults had on average 18% ± 9% (mean ± SD) lower energy intake than the younger adults.

Heterogeneity was not affected by introducing a maximum age of the younger and a minimum age of the older age groups (*I*^2^ = 78%, *n* = 6620 subjects, *p* < 0.001); *i.e.*, after excluding studies in which the mean age or the maximum age, when age was reported as a range, of the younger adult group was >40 years old (studies excluded: mean age of 61 [56]; age range of 30–49 [43], 42–54 [77], 20–64 [78]) and after excluding studies in which the mean age or the minimum age, when age was reported as a range, of the older adult group was <65 years old (studies excluded: mean age of 55 [28], 57 [41], 59 [45], 62 [73], 63 [35] years; age range of 36–53 [31], 50–69 [43]). In the studies included in this sensitivity analysis, energy intake was less in the older than the younger adults with a SMD of −0.87 (95% CI: −1.03 to −0.72). As a group, the older adults (*n* = 2992) had on average 19% ± 9% lower energy intake than the younger (*n* = 3628) adults.

Heterogeneity was not affected by excluding the “small-intestinal” studies, *i.e.*, subjects were intubated with a catheter to deliver the nutrients directly into the small intestine (*I*^2^ = 77%, *n* = 7617, *p* < 0.001; three studies excluded [23,47,48]). In the studies included in this sensitivity analysis, energy intake was less in the older than the younger adults with a SMD of −0.77 (95% CI: −0.90 to −0.63). As a group, the older adults (*n* = 3540) had on average 17% ± 9% lower energy intake than the younger (*n* = 4077) adults.

Heterogeneity was not affected by excluding the “larger” studies, *i.e.*, with >100 subjects per age group (*I*^2^ = 59%, *n* = 2432, *p* < 0.001; four studies excluded [36,43,44,81]). In the studies included in this sensitivity analysis, energy intake was less in the older than the younger adults with a SMD of −0.77 (95% CI: −0.92 to −0.63). As a group, the older adults (*n* = 1212) had on average 18% ± 9% lower energy intake than the younger (*n* = 1220) adults.

In 1555 females (17 studies [28,31,39,43,45,46,47,52,56,63,67,69,70,71,74,78,79]), energy intake after overnight fasting was less in older (*n* = 763) than younger participants (*n* = 792), with a SMD of −0.70 (95% CI: −0.95 to −0.45) and significant heterogeneity (*I*^2^ = 73%, *p* < 0.001). As a group, the older females (1559 kcal) had on average 16% ± 9% lower energy intake than the younger females (1844 kcal).

In 2030 males (28 studies [20,23,30,31,33,35,39,41,43,46,47,49,50,52,55,56,57,58,59,60,71,72,73,74,76,77,78,79]), energy intake after overnight fasting was less in older (*n* = 1045) than younger participants (*n* = 985), with a SMD of −0.95 (95% CI: −1.20 to −0.75) and significant heterogeneity (*I*^2^ = 73%, *p* < 0.001). As a group, the older males (2033 kcal) had on average 18% ± 10% lower energy intake than the younger males (2486 kcal).

Within an individual study, energy intake was significantly less in older than younger adults in five of ten studies that determined energy intake of a single *ad libitum* buffet-style meal after overnight fasting at the research facility [47,48,60,74,75], six of seven studies which determined energy intake by provided food items during a prolonged period [21,58,59,76,79,80], and 24 of 37 studies which determined energy intake by weighed-food records, 24-h food intake recalls, or food frequency questionnaires [20,26,33,39,42,46,47,48,50,51,52,54,55,56,63,67,68,69,70,71,72,73,77,78] (Figure 2). There were no studies in which energy intake after overnight fasting was significantly higher in older than younger adults.

#### 3.1.2. Energy Intake of a Buffet-Style Meal

In the subgroup of 311 subjects in which energy intake was measured of a single *ad libitum* buffet-style meal at the research facility after overnight fasting [23,47,48,57,60,68,69,74,75,81], energy intake was less in the older than the younger adults, with a SMD of −0.89 (95% CI: −1.42 to −0.36) (Figure 2) and significant heterogeneity (*I*^2^ = 77%, *p* < 0.001). As a group, the older adults (*n* = 145, energy intake of 819 kcal/meal) had on average 20% ± 15% (~218 kcal/meal) lower energy intake of a buffet-style meal after overnight fasting than the younger adults (*n* = 166, energy intake of 1037 kcal/meal).

In the subgroup of 266 subjects in which energy intake was measured of a buffet-style meal during postprandial conditions [23,33,44,49,60,68,69,75,81], energy intake was less in the older than the younger adults, with a SMD of −0.37 (95% CI: −0.65 to −0.10) (Figure 2) and no significant heterogeneity (*I*^2^ = 15%, *p* = 0.31). As a group the older adults (*n* = 126, energy intake of 691 kcal/meal) had on average 16% ± 20% (~122 kcal/meal) lower energy intake of a buffet-style meal in a postprandial state than the younger adults (*n* = 140, energy intake of 814 kcal/meal).

In the subgroup of 203 subjects in which energy intake was measured of a buffet-style meal both after overnight fasting and in a postprandial state [23,60,68,69,75,81], energy intake decreased less in the older adults (decrease in energy intake of on average 10% or ~79 kcal from 798 kcal after overnight fasting to 719 kcal in the postprandial state) than in the younger adults (decrease in energy intake of on average 21% or ~212 kcal from 1000 kcal after overnight fasting to 788 kcal in the postprandial state).

#### 3.1.3. Energy Intake during a Prolonged Period

In the subgroup of 339 subjects in which energy intake from provided food items was measured during a prolonged period (~4 days–2 weeks) [21,53,58,59,76,79,80], energy intake was less in the older than the younger adults, with a SMD of −1.09 (95% CI: −1.42 to −0.76) (Figure 2) and non-significant heterogeneity (*I*^2^ = 42%, *p* = 0.11). As a group, the older adults (*n* = 163, energy intake of 2241 kcal/24 h) had on average 19% ± 6% (~546 kcal/24 h) lower energy intake during a prolonged period than the younger adults (*n* = 176, energy intake of 2787 kcal/24 h).

#### 3.1.4. Energy Intake of Weighed-Food Records, 24-h Food Intake Recalls, or Food Frequency Questionnaires

In the subgroup of 7035 subjects in which energy intake was measured using weighed food records [20,28,29,30,31,34,35,39,40,41,43,45,46,47,48,49,50,51,52,55,56,63,68,69,70,72,73,74,75,78], 24-h food intake recall [26,27,42,54,71,77], or food frequency questionnaires [67], energy intake was less in the older than the younger adults with a SMD of −0.69 (95% CI: −0.84 to −0.55) (Figure 2) and significant heterogeneity (*I*^2^ = 77%, *p* < 0.001). As a group the older adults (*n* = 3266, energy intake of 1969 kcal/24 h) had on average 17% ± 8% (~407 kcal/24 h) lower energy intake than the younger adults (*n* = 3769, energy intake of 2376 kcal/24 h).

In the subgroup of 4311 subjects, in which energy intake was measured using weighed food records energy intake was less in the older than the younger adults with a SMD of −0.63 (95% CI: −0.77 to −0.49) and significant heterogeneity (*I*^2^ = 49%, *p* = 0.001).

In the subgroup of 2530 subjects, in which energy intake was measured using 24-h food intake recalls energy intake was less in the older than the younger adults with a SMD of −0.63 (95% CI: −0.77 to −0.49) and significant heterogeneity (*I*^2^ = 92%, *p* = 0.001).

### 3.2. Effect of Age on Appetite

#### 3.2.1. Hunger

Twenty studies (561 subjects) reported hunger after overnight fasting [23,32,33,36,37,38,47,48,49,57,60,61,62,64,65,66,68,69,74,75]. Twelve of these studies (344 subjects) evaluated hunger also after nutrient ingestion (60 min after oral nutrient consumption/start of the small intestinal nutrient infusion; *i.e.*, a time point which was reported in the majority of the studies [23,32,36,37,38,49,65,66,68,69] (two studies did not measure appetite up to 60 min and, therefore, the data of 30 min [75] and 15 min [60] were included); 10 studies (98 subjects) after oral mixed macronutrient (protein, carbohydrate and fat) consumption [32,36,37,38,60,65,66,68,69,75] and two studies (46 subjects) during intraduodenal infusion of protein [23] or fat [49]. All studies reporting hunger were intervention studies, 14 crossover [23,33,36,47,48,49,57,60,61,62,68,69,74,75] and six non-controlled studies [32,37,38,64,65,66],

Nine studies were conducted in Australia [23,32,33,47,48,49,57,68,69], six in Europe [36,37,38,64,65,66], and five in the United States [60,61,62,74,75]. The largest study included 54 subjects [75] and the smallest 10 subjects [57]. The mean age of the youngest group within a study was 22 years (the older group in that study had a mean age of 77 years) and the oldest group 81 years (the young group in that study had a mean age of 38 years) [65].

**Figure 3:**
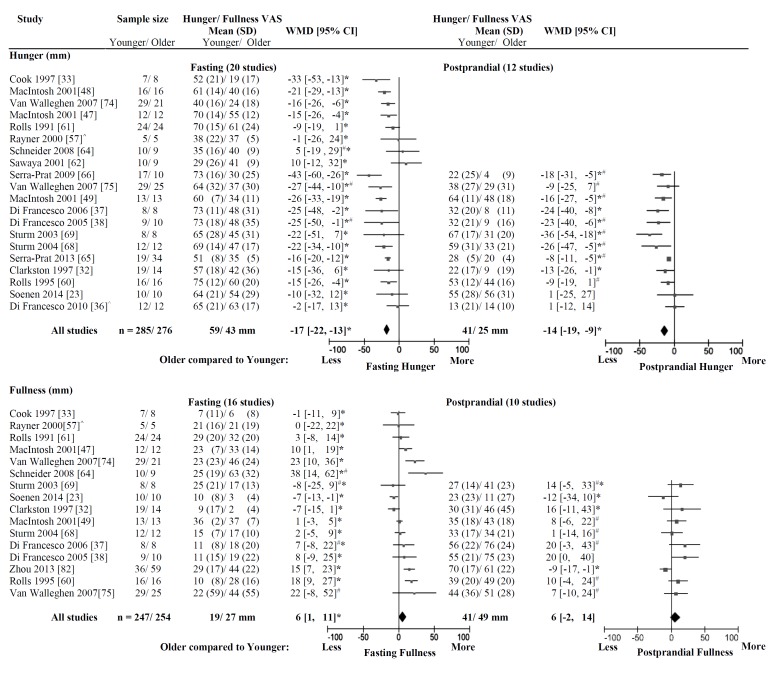
Appetite. Mean ± SD of appetite (hunger and fullness; Visual Analogue Scale (VAS; mm)) after overnight fasting and in a postprandial state and a plot of the weighted mean difference (WMD; mm) of appetite in older compared with younger subjects with the DerSimonian and Laird random-effect model. The horizontal lines denote the 95% confidence interval; ▀ point estimates (the size of the square corresponds to its weight); ♦ the pooled estimate of the age effect. Older compared to younger adults were less hungry (WMD: −17 mm (95% CI −22 to −13 mm), *I*^2^ = 52%, *p* = 0.004) and more full (WMD: 6 mm 95% CI 1 to 11 mm, *I*^2^ = 76%, *p* < 0.001) after overnight fasting and less hungry (WMD: −14 mm (95% CI −19 to −9 mm), *I*^2^ = 53%, *p* = 0.01) in a postprandial state, whereas fullness was comparable (WMD: 6 mm (95% CI −2 to 14 mm), *I*^2^ = 54%, *p =* 0.02). * *p* < 0.05 appetite (hunger/fullness) significantly different in older than younger adults within the study; ^#^ data were derived from a figure of the original publication; ^^^ data were provided by the investigators by e-mail upon request.

Hunger, measured after overnight fasting [23,32,33,36,37,38,47,48,49,57,60,61,62,64,65,66,68,69,74,75], was less in the older (*n* = 285, 74 years, 72 kg, 25 kg/m^2^) than the younger adults (*n* = 276, 27 years, 69 kg, 24 kg/m^2^), with a weighted mean difference (WMD) of −17 mm (95% CI: −22 to −13 mm) (Figure 3) and significant heterogeneity (*I*^2^ = 52%, *p* = 0.004). Heterogeneity was not significant when the “small-intestinal” studies were excluded [23,33,47,48,49] (*I*^2^ =51%, *n* = 444 subjects, *p* = 0.01). As a group, the older adults (43 mm) had on average 25% ± 24% (~16 ± 13 mm) lower hunger after overnight fasting than the younger adults (59 mm).

Hunger, measured in a postprandial state [23,32,36,37,38,49,60,65,66,68,69,75], was less in the older than the younger adults, with a WMD of −14 mm (95% CI: −19 to −9 mm) (Figure 3) and significant heterogeneity (*I*^2^ = 53%, *p* = 0.01). As a group, the older adults had on average 39% ± 30% (~15 ± 11 mm) lower hunger in a postprandial state than the younger adults. In the group of 344 subjects, hunger was decreased by 26 mm from 66 mm after overnight fasting to 40 mm in a postprandial state in the younger adults and by 20 mm from 45 mm after overnight fasting to 25 mm in a postprandial state in the older adults.

Within an individual study, hunger was significantly less in older than younger adults in 12 [33,37,38,47,48,49,57,60,65,66,74,75] of 20 studies after overnight fasting and eight [32,37,38,49,65,66,68,69] of 12 studies in a postprandial state. There were no studies in which hunger was significantly higher in older than younger adults.

#### 3.2.2. Fullness

Sixteen studies (501 subjects) reported fullness after overnight fasting [23,32,33,37,38,47,49,57,60,61,64,68,69,74,75,82]. Ten of these studies (335 subjects) evaluated fullness also after nutrient ingestion; eight studies after oral mixed macronutrient consumption [32,37,38,60,68,69,75,82]; and two studies during intraduodenal infusion of protein [23] or fat [49]. All studies reporting fullness were intervention studies, 12 crossover [23,33,47,49,57,60,61,68,69,74,75,82] and four non-controlled studies [32,37,38,64].

Eight studies were conducted in Australia [23,32,33,47,49,57,68,69], four in the United States [60,61,74,75], three in Europe [37,38,64], and one in Asia [82]. The largest study included 95 subjects [82] and the smallest 10 subjects [57]. The mean age of the youngest group was 22 years and 77 years of the oldest group [69].

Fullness, measured after overnight fasting, was greater in the older (*n* = 254, 73 years, 71 kg, 25 kg/m^2^) than the younger adults (*n* = 247, 26 years, 67 kg, 23 kg/m^2^), with a WMD of 6 mm (95% CI: 1 to 11 mm) and significant heterogeneity (*I*^2^ = 76%, *p* < 0.001; Figure 3). Heterogeneity was not affected by excluding the “small-intestinal” studies (*I*^2^ = 73%, *n* = 416 subjects, *p* < 0.001; four studies excluded [23,33,47,49]). As a group the older adults (27 mm) had on average 37% ± 73% (~8 ± 13 mm) higher fullness after overnight fasting than the younger adults (19 mm) adults.

Fullness, measured in a postprandial state, was not significantly different between the older and the younger subjects with a WMD of 6 mm (95% CI: −2 to 14 mm) and significant heterogeneity (*I*^2^ = 54%, *p =* 0.02). In the group of 335 subjects, fullness was increased by 23 mm from 18 mm after overnight fasting to 41 mm in a postprandial state in the younger adults and by 26 mm from 23 mm after overnight fasting to 49 mm in a postprandial state in the older adults.

Heterogeneity decreased by introducing a maximum age of the younger and a minimum age of the older age groups (*I*^2^ = 0%, *n* = 240 subjects, *p* = 0.51); *i.e.*, after excluding studies in which the mean age or the minimum age, when age was reported as a range, of the older adult group was <65 years old (one study excluded: range of 50–59 years [82])—there were no studies in which the mean age or the maximum age, when age was reported as a range, of the younger adult group was >40 years old. In the studies included in this sensitivity analysis, fullness was significantly different between the older and the younger adults with a WMD of 9 mm (95% CI: 2 to 14). As a group, the older adults had on average 21% ± 32% (~9 ± 10 mm) higher fullness in a postprandial state than the younger adults.

Within an individual study, fullness was greater in older than younger adults in six [23,47,60,64,74,82] of 16 studies after overnight fasting. In contrast, fullness was less in older than younger adults in one [23] of 16 studies after overnight fasting and one [82] of 10 studies in a postprandial state.

## 4. Discussion

This meta-analysis examined the effect of ageing on appetite and energy intake in adults, including data from >7500 subjects on energy intake and >500 subjects on appetite derived from 59 studies. Energy intake was less in healthy older (~70 years) than younger (~26 years) adults. The calculated reduction fell into quite a narrow range at 16%–20%, despite studies being done in the fasting and fed state, and energy intake being calculated by a variety of methods, including intake at an acute study meal, during prolonged periods or using weighed food records, 24-h food intake recalls, and food frequency questionnaires, *i.e.*, a robust finding regardless of the method of intake evaluation. The results of this analysis show that older people (~73 years) feel less hungry than younger adults (~26 years), both fasting (25%) and after they have consumed some food (39%), and also feel more full in the fasting state (37%). These age-related differences are substantial, and likely to be a major cause of the reduced energy intake by older people.

Our results indicate a reduction in energy intake of approximately 20% between the ages of 26 and 70 years, *i.e.*, about 0.5% per year. This is consistent with previous reports of reduced energy intake of approximately 30% between the ages of 20 and 80 years [5,79], and with the results of individual prospective studies. For example, a seven-year New Mexico longitudinal study of 156 persons aged 64–91 years, reported a decrease of 19 kcal/day/year in women and 25 kcal/day/year in men [3], while a Swedish longitudinal study of 98 people found an even greater decline of energy intake of 610 kcal/day in men and 440 kcal/day in women, between the ages of 70 and 76 years [4]. A population-based study indicated that older people aged 60–74 years consume ~500–700 kcal/day less than their younger counterparts aged 20–39 years [5]. Our gender analyses indicated that energy intake was less in both older than younger males (18%) and females (16%), to a similar extent in both sexes.

The regulation of energy intake may be diminished in the elderly. Older subjects have a reduced suppression of energy intake after oral [60], or small intestinal nutrient [23], ingestion. In this meta-analysis in the subgroup of 203 subjects (six studies [23,60,68,69,75,81]), in which energy intake was measured during a single *ad libitum* buffet-style meal at the research facility both after overnight fasting and in the postprandial state, energy intake decreased on average 11% less in the older than young adults. Older people do not show the ability to regulate food intake after prolonged over- or under-feeding as young individuals [53,59]. This indicates that after an anorectic insult (for example, major surgery), older people are likely to take longer than young adults to regain the weight lost, remain undernourished longer, and be more susceptible to subsequent superimposed illnesses, such as infections.

Our results indicate a reduction in hunger of approximately 25% and increase in fullness of approximately 35% between the ages of 27 and 74 years, *i.e.*, changes of about 0.5% per year for hunger and about 0.7% per year for fullness, respectively. Scores for appetite are predictive of energy intake in both healthy young and older subjects [84]. Appetite and energy intake are dependent on the precise co-ordination of interrelated “intragastric” (*i.e.*, gastric emptying [85], antral area and motility (the distal stomach) [68,85], and plasma ghrelin concentrations [69,86,87,88]) and “small intestinal” mechanisms (pyloric motility [89] and gut hormone secretion including cholecystokinin (CCK) [86], glucagon-like polypeptide-1 (GLP-1) [90], peptide tyrosine tyrosine (PYY) and gastric inhibitory polypeptide (GIP)). These gastrointestinal mechanisms affecting appetite and energy intake are modulated by ageing [91]. Healthy older people, as a group, have slightly slower gastric emptying [32] mediated by increased pyloric motility [33,34,68,69], greater gastric antral area [68], decreased perception of gastric distension [57], lower plasma ghrelin [92] and higher CCK concentrations than young adults, differences that all favour reductions in appetite and energy intake. Ageing is associated with insulin resistance and impaired glucose tolerance [93] which may be influenced by changes in small intestinal hormone (GLP-1, GIP) secretion [94]. In addition, thyroid hormone concentrations are known to change with ageing which may be regarded as a physiologic process that can affect appetite [95,96]. There may be a decrease in appetite-stimulating free thyroid hormones with increasing age in men [97]. Serum thyroid stimulating hormone (TSH) concentrations may be higher and free thyroid hormones lower in older, when compared to younger, men [98]. Older people have an increased prevalence of both hypo- (up to 5%) and hyperthyroidism (0.5%–3%) than younger patients. In the elderly the symptoms of both conditions can overlap with other age-related diseases (e.g., unexplained, weight loss, anorexia, weakness, fatigue, depression, constipation) [98]. The senses of smell and taste deteriorate with age [99], leading to a reduced capacity to enjoy food and develop sensory-specific satiety, [61] the normal decline in pleasantness of the taste of a particular food after it has been consumed, leading to a decrease in its consumption and a tendency to shift consumption to other food choices during a meal. Age-related reduction in sensory-specific satiety favours a less varied, more monotonous diet, and the development of micronutrient deficiencies.

Physiological anorexia and seemingly minor weight loss predisposes to the development of pathological under-nutrition, cachexia and adverse effects [100] and is, accordingly, associated with increased morbidity and mortality. For example, in a large study of community-dwelling Americans aged 65 years or older, weight loss in excess of 5% body weight over three years occurred in 17% and was associated with a 70% increase in mortality, irrespective of the initial weight, whereas weight stability and weight gain were not associated with increased mortality [7]. Not uncommonly, pathological anorexia and weight loss are superimposed on the “physiological anorexia of ageing” [19]. This can be the result of a variety of conditions that become more frequent with age, including acute and chronic medical conditions (gastrointestinal disease, malabsorption syndromes, infection, hypermetabolism, micronutrient deficiencies, increased energy requirements), medications (which may cause malabsorption of nutrients, gastrointestinal symptoms, and loss of appetite), psychological factors (depression, dementia and Alzheimer’s disease, and bereavement), social factors (poverty, difficulties with shopping, meal preparation and self-feeding, living alone, social isolation and loneliness) and physical factors (poor dentition leading to problems with chewing, immobility (stroke), Parkinson disease, and impaired vision). Because the majority of these factors are at least partly responsive to treatment, their recognition is important. For example, increased cytokine levels, due to the stress of ageing *per se*, or the amplified stressful effects of other pathologies, may provide an explanation for some of the decline in appetite and energy intake in older people [101]. Increased cortisol and catecholamines stimulate the release of interleukin 6 and tumour necrosis factor alpha [102].

Although only a limited number of studies have examined the effects of undernutrition on appetite and energy intake, there is evidence of substantial differences between undernourished and well-nourished older people, which may potentially result from being undernourished and/or contribute to the undernourished state [65,66,69]. Undernourished older adults had significantly reduced hunger in the fasted state and in the postprandial state’ and significant greater fullness in the fasted state when compared to healthy older [65] and young adults [65,69]. In undernourished older women energy intake was not suppressed by a mixed-nutrient preload, unlike in well-nourished older and young women [69]. In another study of undernourished older subjects, concentrations of CCK were higher than in well-nourished older subjects [103], suggesting that increased CCK activity may be a cause of undernutrition in older people, and/or act to perpetuate it.

Limitations of the meta-analysis are that we used a single database and that there is variability in study design and characteristics indicated by high heterogeneity—we performed *a-priori* determined meta-analyses depending on the method used to determine energy intake and sensitivity analyses when possible, *i.e.*, effect of sex and age, and observed that the effects of ageing on appetite and energy intake were comparable in these analyses.

## 5. Conclusions

In summary, this meta-analysis of 59 studies supports previous reports that appetite and energy intake are reduced in healthy older compared with younger adults, with a 16%–20% lower energy intake, 25%–39% lower hunger and 37% more fullness in those aged on average 70–74 years compared to 26–27 years, a robust finding regardless of the method of intake evaluation. These age-related differences in healthy adults are consistent with a reduction of food intake with ageing, *i.e.*, a physiological anorexia of ageing. The reduction in energy intake in this analysis equates to approximately 0.5% per year of increasing age, and is likely to contribute to loss of weight in older people and the development of pathological under-nutrition in predisposed older people.
